# Spatial and Temporal Boundaries of Remote Areas in the Initial Management of Out-of-Hospital Cardiac Arrest

**DOI:** 10.7759/cureus.78427

**Published:** 2025-02-03

**Authors:** Mihaela Budimski Soldat, Srdjan S Nikolovski, Zoran Fiser, Goran Provci, Ankica Vasic, Zlatko Babic, Deze Babinski, Erika Terek, Ivana Kicivoj, Jelena Tijanic, Branislav Martinovic, Aleksandra Lazic, Suzana S Randjelovic, Kornelija Jaksic Horvat, Violetta Raffay

**Affiliations:** 1 Emergency Medicine, Municipality Primary Health Care Center Subotica, Subotica, SRB; 2 Pathology and Laboratory Medicine, Cardiovascular Research Institute, Loyola University Chicago, Maywood, USA; 3 Nursing, Municipality Emergency Medical Center Novi Sad, Novi Sad, SRB; 4 Emergency Medicine, Municipality Primary Health Care Center Backa Topola, Backa Topola, SRB; 5 Emergency Medicine, Municipality Primary Health Care Center Becej, Becej, SRB; 6 Emergency Medicine, Municipality Primary Health Care Center Vrbas, Vrbas, SRB; 7 Emergency Medicine, Municipality Primary Health Care Center Kanjiza, Kanjiza, SRB; 8 Emergency Medicine, Municipality Primary Health Care Center Senta, Senta, SRB; 9 Emergency Medicine, Municipality Primary Health Care Center Inđija, Inđija, SRB; 10 Emergency Medicine, Municipality Primary Health Care Center Kragujevac, Kragujevac, SRB; 11 Emergency Medicine, Municipality Primary Health Care Center Ruma, Ruma, SRB; 12 Emergency Medicine, Resuscitation Council of Serbia, Novi Sad, SRB; 13 Emergency Medicine, University Clinical Center Kragujevac, Kragujevac, SRB; 14 Medicine, School of Medicine, European University Cyprus, Nicosia, CYP

**Keywords:** bystander, cardiac arrest, cardiopulmonary resuscitation, dc shock, emergency medical service, out-of-hospital cardiac arrest, return of spontaneous circulation, ventricular fibrillation

## Abstract

Introduction: Besides well-described patient-related and event-related factors, time intervals during the initial management of out-of-hospital cardiac arrest (OHCA) and distance from the emergency call and collapse location are additional influencing elements usually underrepresented by the widely applied prognostic models. The aim of this study is to analyze the influence of time and distance as crucial factors on the success of pre-hospital care of patients with OHCA, as well as the influence of other variables defined by the EuReCa Study protocol, observed during the study period, on the positive pre-hospital outcomes.

Methods: According to the EuReCa Study protocol accepted by the EuReCa_Serbia protocol, the data on all cases of adult patients with witnessed cardiac-cause OHCA receiving bystander cardiopulmonary resuscitation (CPR) measures were prospectively collected during the period October 1, 2014 to December 31, 2023, and analyzed to compare the degree of influence of different patient- and OHCA-related predictors, including time- and distance-related factors, on initial OHCA outcomes - first recorded heart rhythm, return of spontaneous circulation on scene (any ROSC), and survival to hospital admission.

Results: During the follow-up period, 2,261 cases were registered. Female sex and OHCA location in the patient’s residence were the main negative independent predictors, while full CPR measures were the main positive independent predictor of shockable initial heart rhythm. OHCA occurring in a public building, full CPR measures, and direct current (DC) shock delivery were the main positive predictors of any ROSC. Population size larger than 100,000 inhabitants and OHCA location in the patient’s residence were the main negatives, while OHCA location on the street was the main positive predictor of survival to hospital admission. Emergency medical service response time in minutes and distance from the emergency call to the collapse location in kilometers were significant predictors of both shockable initial heart rhythm (p < 0.001, OR = 0.928, 95% CI = 0.889-0.967 and p < 0.001, OR = 0.910, 95% CI = 0.864-0.959, respectively) and any ROSC (p < 0.001, OR = 0.898, 95% CI = 0.855-0.943 and p < 0.001, OR = 0.874, 95% CI = 0.823-0.930, respectively).

Conclusion: Location, patient sex, and bystander CPR type play an important role in predicting shockable initial rhythm. The same factors, except the patient's sex, predicted any ROSC, while the main predictors of survival to hospital admission are population size and OHCA location. Both shorter emergency medical service response time and a shorter distance are significantly associated with a higher rate of shockable initial rhythm and any ROSC.

## Introduction

Heart diseases are the main cause of out-of-hospital cardiac arrest (OHCA), which is the leading global cause of mortality. Early positive on-scene and hospital admission outcomes are significant factors that influence overall survival, in-hospital treatment success, and complete recovery, as well as the preservation of life quality. There is a relative paucity of data on factors, particularly time and distance between emergency call and collapse location, that have a predictive influence on return of spontaneous circulation on scene (any ROSC) and survival to admission and discharge in patients with cardiac-cause OHCA where bystander initiates cardiopulmonary resuscitation (CPR).

Positive pre-hospital outcomes have been well-documented in the literature to be strongly correlated with survival to hospital discharge and long-term survival [1,2]. Previous studies have also demonstrated that OHCA cases with cardiac etiology exhibit significantly better positive outcomes and survival rates when compared to non-cardiac etiology cases [3].

Underlying cardiologic or presumed cardiologic condition is the most common etiology of OHCA [4,5]. This suggests a basis for a distinct analysis of OHCA of cardiac or presumed cardiac etiology compared to other causes of OHCA, particularly OHCA resulting from trauma.

In a scenario involving Utstein events, defined as cases where bystander-witnessed cardiac-cause OHCA events with detected shockable initial heart rhythm occur, which have a high likelihood of achieving return of spontaneous circulation at the collapse location (any ROSC) [1], it is crucial to identify factors beyond those outlined in the Utstein event criteria that impact the primary pre-hospital positive outcomes in these patients - any ROSC and survival to hospital admission.

The national registry of OHCA in Serbia (EuReCa_Serbia) was established in 2014 when the collection of OHCA-related epidemiological data was commenced in the organization of the Serbian Resuscitation Council.

Considering the assumption that time is the most critical factor, followed by distance, and that even in urban areas, there may be “remote areas” where high-quality and timely medical attention with positive outcomes is not guaranteed, the aim of this study is to analyze and shed a light on the influence of time and distance as crucial factors on the success of pre-hospital care of patients with OHCA and to define their values significant for survival in the conditions present on the analyzed regions in Serbia. This study also considers and analyzes the influence of other variables defined by the EuReCa Study, observed during the analyzed period, on positive pre-hospital outcomes in these patients. The results of this analysis could contribute to improving the organization and utilization of emergency medical system resources.

## Materials and methods

Patient-related and OHCA event-related data were prospectively collected by randomly selected emergency medical service centers in Serbia for 2,261 cardiac-cause OHCA events during the period October 1, 2014, to December 31, 2023, according to the EuReCa_Serbia protocol, which accepted the EuReCa Study protocol (U.S. National Library of Medicine’s Clinical Trials Registry number NCT02236819). The analysis was performed on cardiac-cause OHCA events in adult patients where bystander-initiated CPR measures were applied. The occurrence of initially observed shockable heart rhythm (ventricular tachycardia or ventricular fibrillation) on-scene and any ROSC, as well as the return of spontaneous circulation (ROSC) on hospital admission, were analyzed as the outcome variables. Demographic patient-related data and preceding event-related data were observed as factors with potential influence on the outcome variables.

Data were collected as defined by the EuReCa Study protocol and according to its dataset as a component of the project's documentation. EuReCa_Serbia study, as a part of the European Resuscitation Council's EuReCa Study, was conducted in 18 research centers. Data collection was conducted by individual researchers and entered into a unique electronic database by the principal investigator of each research center. During the process of entering the data, principal investigators conducted a logical control of the collected data. In case of missing or inaccurate entries, the whole data for individual patients was sent for review to the researchers of the corresponding research centers. The second data control level was conducted by the principal investigator of the EuReCa_Serbia project who checked all already entered data for completeness and the fact whether the entered data correctly answered the questions defined by the EuReCa_Serbia project's dataset. In situations where the entered information did not represent the answer to the defined question, the choice "unknown" was selected, which is assigned for all missing data by the EuReCa Study dataset.

The procedures of this study were conducted in accordance with the regulations set forth by the Helsinki Declaration of the World Medical Association, updated in 2013, and approved by the Ethics Committee of the Serbian Resuscitation Council (approval number: A-034-150614-2014) on June 15, 2014, following the EuReCa Study protocol, which exempted the collection of participants’ informed consent.

Descriptively, continuous variables were presented as median values and interquartile range (IQR), while categorical variables were shown as frequencies and percentages. The distribution of continuous variables was assessed using the Kolmogorov-Smirnov test with Lilliefors correction. Association analysis was conducted using the chi-square test and Mann-Whitney U test. Causality was evaluated with univariable and multivariable binary logistic regression. Receiver operating characteristic analysis was performed to determine the cut-off values of emergency medical service (EMS) response time (defined as the time interval from initiation of the emergency call to the EMS team's arrival at the collapse location) and EMS response distance (distance from the location of emergency call acceptance to the collapse location). The statistical significance level was set at 0.05. The analysis was performed using SPSS for Windows v27 (IBM Corp., Armonk, NY) and GraphPad Prism v10 for Windows (GraphPad Software Inc., San Diego, CA).

## Results

During the period from October 1, 2014, to December 31, 2023, a total of 10,458 OHCA cases were registered, with 6,712 of 10,458 (64.2%) attributable to a cardiac or presumed cardiac condition, which resulted in an annual incidence rate of 38.86 per 100,000.

**Figure 1:**
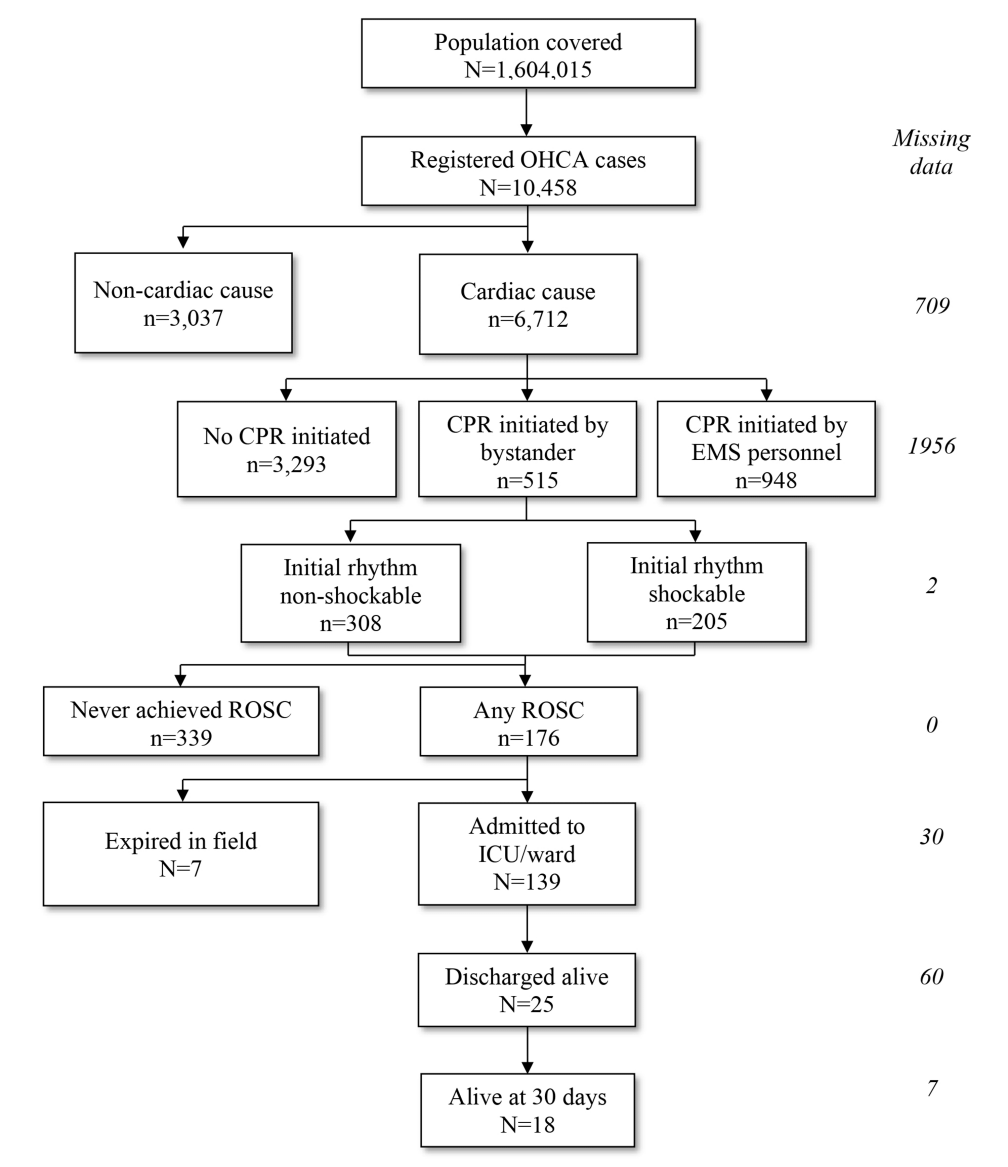
Utstein flowchart. OHCA: out-of-hospital cardiac arrest; CPR: cardiopulmonary resuscitation; EMS: emergency medical service; ROSC: return of spontaneous circulation; ICU: intensive care unit.

Of all cardiac-cause OHCA cases, CPR was initiated by a bystander in 515 of 6,712 cases (7.7%), on which further analysis was performed. In this group of patients, the median patient age was 65 years (IQR = 57-72). The flowchart of data analysis is presented in Figure 1.

The median age of the patients was 65 years (IQR = 57-72), with over half of them (54.2%) being 65 years old or younger. Less than one-third of the patients (31.8%) were female.

The residence as an event location was noted in 331/515 cases (64.3%), and dispatcher assistance was provided in 190/515 cases (36.9%). Compression-only CPR was administered in 199/515 cases (38.6%), full compression and ventilation CPR measures in 180/515 (35.0%), while in the remaining 136 of 515 bystander-CPR cases, information on the type and character of applied measures was missing (26.4%). A shockable first recorded rhythm was detected in 205/515 patients (39.8%), direct current (DC) shock was delivered in 119/515 cases (23.1%), while any ROSC was observed in 176/515 patients (34.2%).

The median EMS response time was 8.0 (IQR 5.0-13.0) minutes, while the median distance from the call-receiving location to the collapse location was 8.0 (4.2-10.4) kilometers.

Factors significantly associated with the occurrence of shockable initial heart rhythm, any ROSC, and survival to hospital admission were the patient's age, as well as the patient’s residence and public building as the collapse location.

Factors significantly associated with the occurrence of shockable initial heart rhythm and any ROSC were the application of cardiac compression-only (CCO) CPR vs. full CPR, as well as the DC shock delivery. Additionally, patient sex and sports facility as the collapse location were additional factors significantly associated with the occurrence of initial shockable heart rhythm, while population size of the sampling region and street as the location of the collapse were additional factors significantly associated with survival to hospital admission (Table 1).

**Table 1:** Association analysis between OHCA-related factors and main pre-hospital outcomes. ROSC: return of spontaneous circulation; CPR: cardiopulmonary resuscitation; DA-CPR: dispatcher-assisted CPR; CCO: cardiac compressions only; DC: direct current.

Variables		Shockable rhythm, N (%)	p	Any ROSC, N (%)	p	Survival to admission, N (%)	p
Population size	≤100,000	83 (40.9)	0.062	57 (32.8)	0.293	50 (36.5)	0.004
	>100,000	120 (59.1)		117 (67.2)		87 (63.5)	
Patient age	≤65 years	140 (68.3)	<0.001	113 (64.2)	<0.001	91 (65.5)	0.016
	>65 years	65 (31.7)		63 (35.8)		48 (34.5)	
Patient sex	Male	150 (73.2)	0.042	121 (68.8)	0.835	97 (69.8)	0.44
	Female	55 (26.8)		55 (31.2)		42 (30.2)	
Location: residence	Yes	112 (54.6)	<0.001	87 (49.4)	<0.001	60 (43.2)	<0.001
	No	93 (45.4)		89 (50.6)		79 (56.8)	
Location: long-term care	Yes	2 (1.0)	>0.999	1 (0.6%)	0.665	1 (0.7)	0.603
	No	203 (99.0)		175 (99.4)		138 (99.3)	
Location: work/office	Yes	6 (2.9)	0.065	4 (2.3)	0.454	4 (2.9)	0.687
	No	199 (97.1)		172 (97.7)		135 (97.1)	
Location: street	Yes	29 (14.1)	0.655	30 (17.0)	0.337	29 (20.9)	<0.001
	No	176 (85.9)		146 (83.0)		110 (79.1)	
Location: public building	Yes	28 (13.7)	<0.001	34 (19.3)	<0.001	34 (24.5)	<0.001
	No	177 (86.3)		142 (80.7)		105 (75.5)	
Location: sports facility	Yes	8 (3.9)	<0.001	5 (2.8)	0.129	3 (2.2)	>0.999
	No	197 (96.1)		171 (97.2)		136 (97.8)	
Location: outpatient hospital	Yes	0 (0.0)	/	0 (0.0)	/	0 (0.0)	/
	No	205 (100.0)		176 (100.0)		139 (100.0)	
DA-CPR	Yes	70 (34.5)	0.221	59 (34.1)	0.249	57 (41.9)	0.068
	No	133 (65.5)		114 (65.9)		79 (58.1)	
CCO vs. full CPR	CCO	66 (42.3)	<0.001	55 (41.0)	<0.001	47 (45.6)	0.272
	Full CPR	90 (57.7)		79 (59.0)		56 (54.4)	
DC shock	/	/	/	62 (35.2)	<0.001	58 (41.7)	0.062
	/	/		114 (64.8)		81 (58.3)	

The predictive potential of the factors showing significant association with main pre-hospital OHCA outcomes was further evaluated by binary logistic regression analysis.

**Figure 2:**
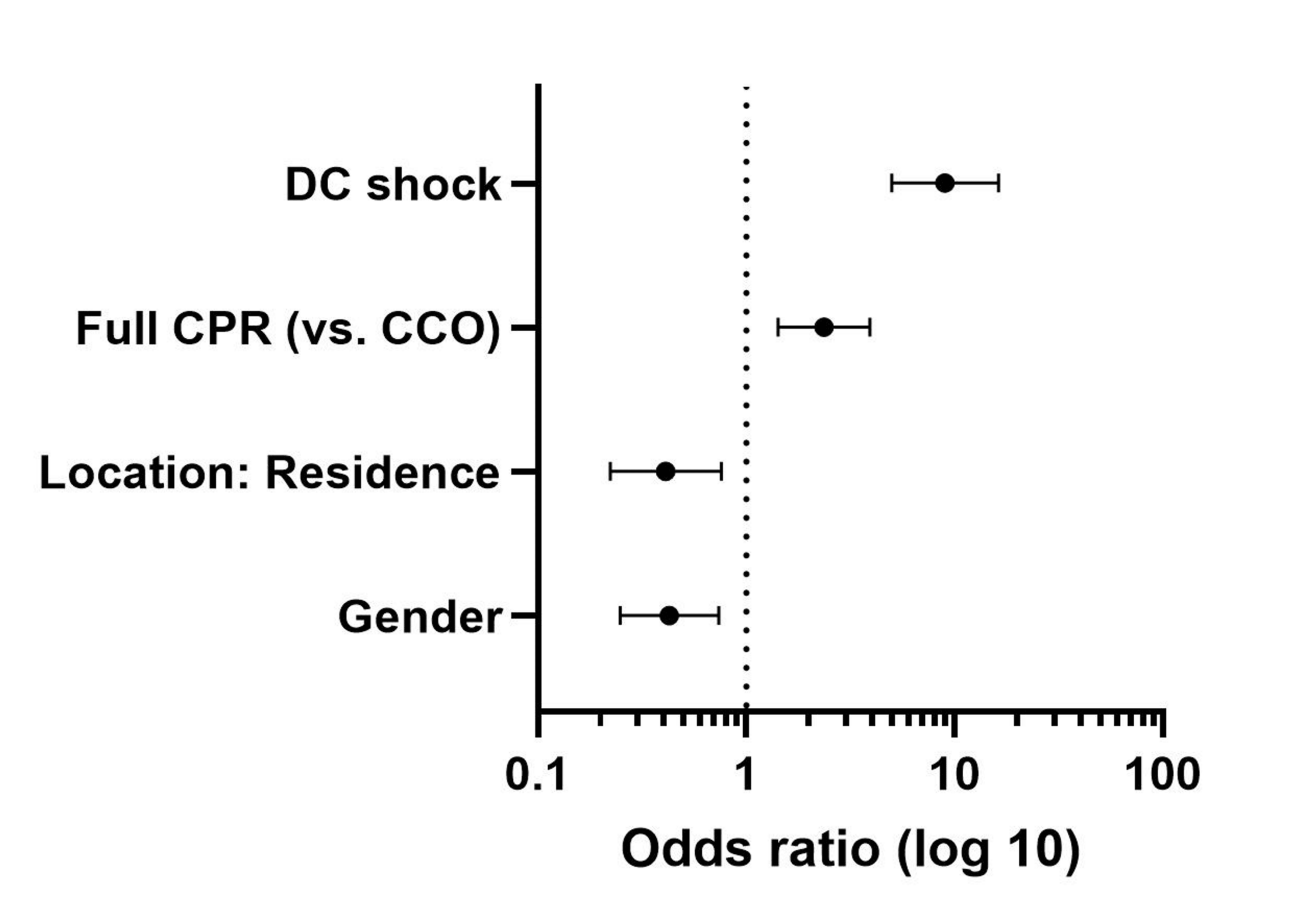
Predictors of shockable initial heart rhythm. DC: direct current; CPR: cardiopulmonary resuscitation; CCO: cardiac compressions only.

**Figure 3:**
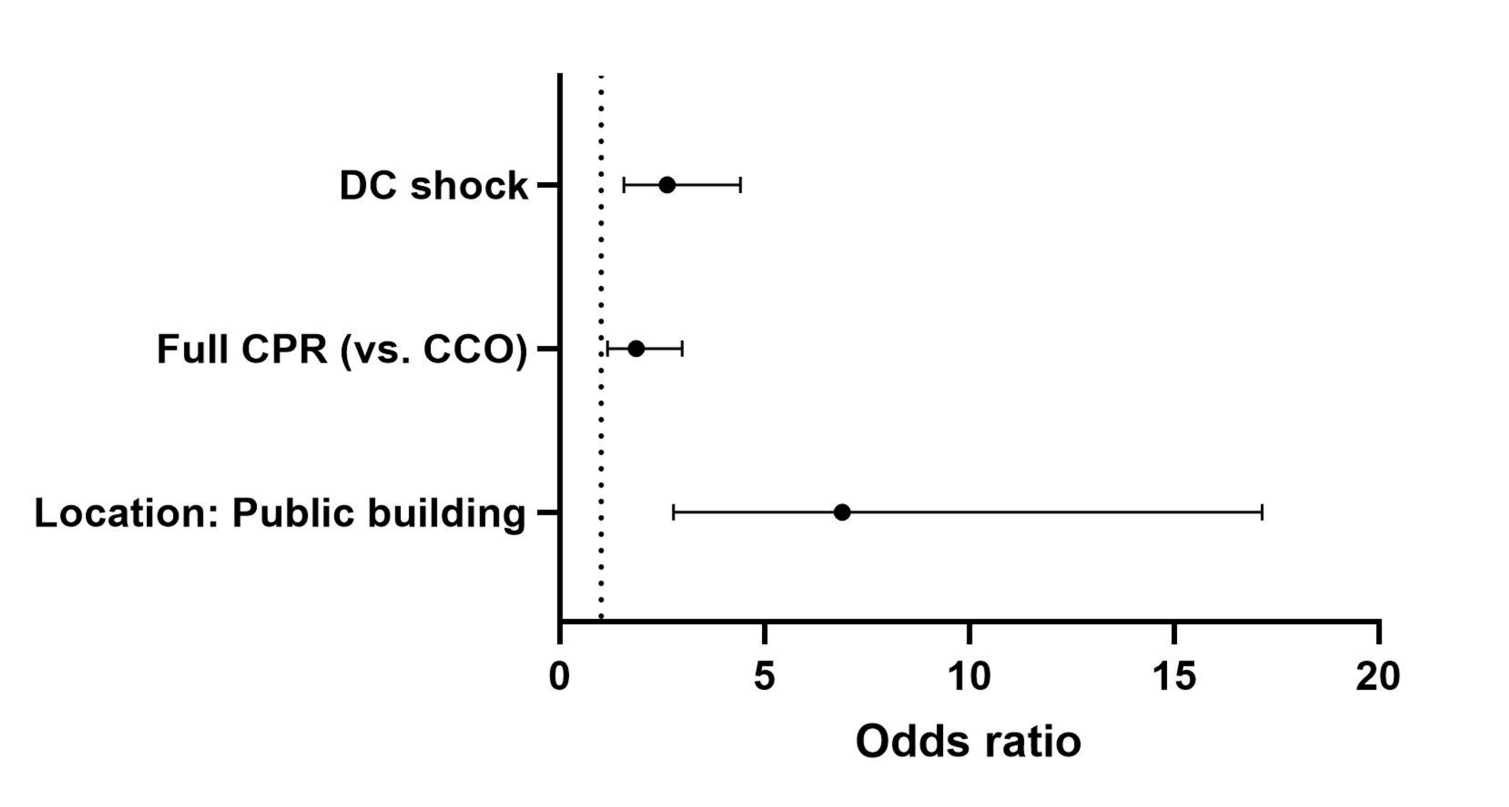
Predictors of survival to admission. DC: direct current; CPR: cardiopulmonary resuscitation; CCO: cardiac compressions only.

**Figure 4:**
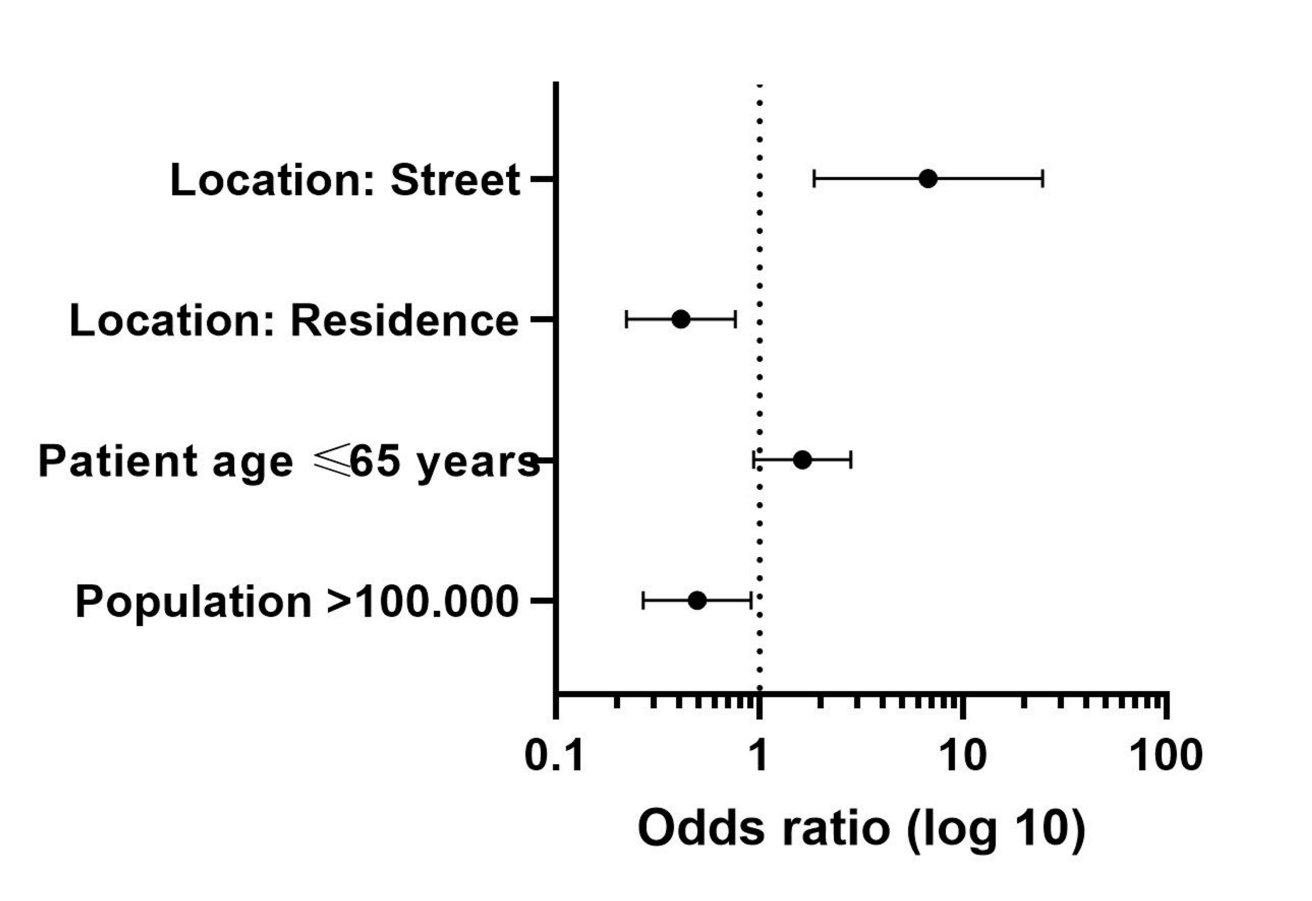
Predictors of survival to admission.

Female sex and OHCA location in the inpatient’s residence were the main negative independent predictors of shockable initial heart rhythm. Applying full CPR measures (compared to CCO) was the main positive predictor of this outcome. OHCA occurring in a public building, applying full CPR measures (compared to CCO), and DC shock delivery were the main positive predictors of any ROSC. Population size larger than 100,000 inhabitants and OHCA location in the patient’s residence were the main negative, while OHCA location on the street was the main positive predictor of survival to hospital admission (Tables 2-4 and Figures 2-4).

**Table 2:** Predictors of shockable initial heart rhythm. OR: odds ratio; CI: confidence interval; CPR: cardiopulmonary resuscitation; CCO: cardiac compressions only; DC: direct current.

Factors	Univariable analysis			Multivariable analysis		
	p	OR	95% CI	p	OR	95% CI
Patient age ≤ 65 years	<0.001	2.688	1.857-3.893	0.325	1.287	0.778-2.128
Female sex	0.042	0.669	0.454-0.986	0.002	0.429	0.249-0.739
Location: residence	<0.001	0.489	0.338-0.708	0.005	0.412	0.223-0.761
Location: public building	<0.001	3.322	1.703-6.480	0.091	2.176	0.883-5.358
Location: sports facility	No data					
Full CPR (vs. CCO)	<0.001	2.022	1.334-3.066	<0.001	2.366	1.424-3.930

**Table 3:** Predictors of any ROSC. ROSC: return of spontaneous circulation; OR: odds ratio; CI: confidence interval; CPR: cardiopulmonary resuscitation; CCO: cardiac compressions only; DC: direct current.

Factors	Univariable analysis			Multivariable analysis		
	p	OR	95% CI	p	OR	95% CI
Patient age ≤ 65 years	0.001	1.869	1.285-2.719	0.316	1.281	0.790-2.078
Location: residence	<0.001	0.381	0.261-0.556	0.152	0.663	0.378-1.164
Location: public building	<0.001	8.779	4.103-18.784	0.000	6.898	2.775-17.146
Full CPR (vs. CCO)	0.001	2.048	1.335-3.142	0.010	1.859	1.159-2.981
DC shock	<0.001	2.691	1.767-4.096	<0.001	2.619	1.556-4.407

**Table 4:** Predictors of survival to admission. OR: odds ratio; CI: confidence interval.

Factors	Univariable analysis			Multivariable analysis		
	p	OR	95% CI	p	OR	95% CI
Population >100,000	0.004	0.439	0.251-0.769	0.024	0.494	0.268-0.911
Patient age ≤ 65 years	0.016	1.836	1.118-3.015	0.086	1.622	0.934-2.818
Location: residence	<0.001	0.232	0.136-0.396	0.005	0.411	0.222-0.760
Location: street	<0.001	4.406	1.855-10.470	0.004	6.748	1.855-24.552
Location: public building	<0.001	13.06	3.898-43.756	0.956	0.948	0.144-6.229

Influence of time and distance to collapse location on pre-hospital outcomes

Both EMS response time in minutes and distance from the call receiving location to the collapse location in kilometers were significantly shorter in cases eventually reaching shockable initial heart rhythm and any ROSC, while no significant difference was observed in terms of survival to hospital admission (Table 5).

**Table 5:** Differences in EMS response time and EMS response distance between observed outcomes. EMS: emergency medical service; IQR: interquartile range; ROSC: return of spontaneous circulation.

	Shockable rhythm	Non-shockable rhythm	p
EMS response time in minutes (Med (IQR))	5.0 (3.0-12.0)	10.0 (6.0-13.0)	<0.01
EMS response distance in kilometers (Med (IQR))	5.6 (2.4-9.6)	8.8 (5.6-10.4)	<0.01
	Any ROSC	No any ROSC	
EMS response time in minutes (Med (IQR))	5.0 (3.0-11.0)	10.0 (6.0-13.0)	<0.01
EMS response distance in kilometers (Med (IQR))	6.0 (2.4-9.6)	8.8 (5.6-11.2)	<0.01
	Admission survival	No admission survival	
EMS response time in minutes (Med (IQR))	8.0 (3.0-13.0)	6.0 (4.0-6.2)	0.790
EMS response distance in kilometers (Med (IQR))	7.2 (2.4-10.4)	4.8 (3.2-5.2)	0.892

EMS response time in minutes and distance from the emergency call receiving location to the collapse location in kilometers were also observed as significant predictors of both shockable initial heart rhythm (p < 0.001, odds ratio (OR) = 0.928, 95% CI = 0.889-0.967 and p < 0.001, OR = 0.910, 95% CI = 0.864-0.959, respectively) and any ROSC (p < 0.001, OR = 0.898, 95% CI = 0.855-0.943 and p < 0.001, OR = 0.874, 95% CI = 0.823-0.930, respectively). However, EMS response time and distance were not significant predictors of survival to hospital admission (p = 0.628, OR = 1.084, 95% CI = 0.783-1.499 and p = 0.628, OR = 1.106, 95% CI = 0.737-1.659, respectively).

The cumulative proportion of any ROSC reached 50% after four minutes of the EMS response time and after 5.6 kilometers of the distance from the emergency call receiving location to the collapse location.

**Figure 5:**
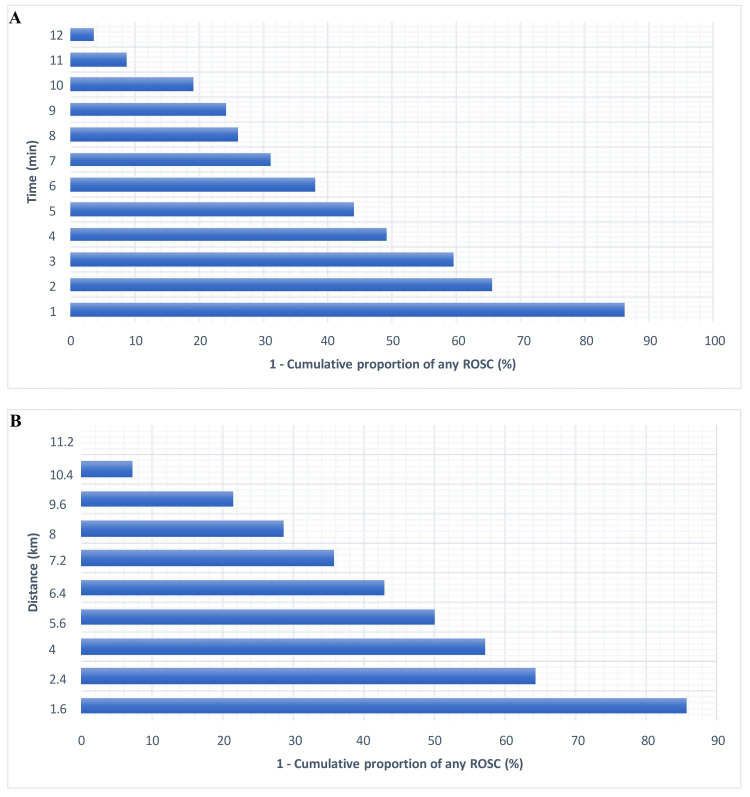
Inverse any ROSC cumulative proportion curve related to the EMS response time (A) and the distance from the emergency call receiving location to the collapse location (B). ROSC: return of spontaneous circulation; EMS: emergency medical service.

No cases of any ROSC were observed when the EMS response time was 20 minutes or longer and when the distance from the emergency call receiving location to the collapse location was 11.2 kilometers or more. Cumulative proportions of achieving any ROSC are presented for each time and distance unit in Figure 5.

The receiver operating curve (ROC) analysis of the EMS response time in function of any ROSC revealed a cut-off value of 4.5 minutes with a sensitivity value of 40.5% and a specificity value of 90.4% (p < 0.001, area under the curve (AUC) = 0.670, 95% CI = 0.607-0.732). The ROC analysis of the distance from the emergency call receiving location to the collapse location in the function of any ROSC indicated a cut-off value of 3.6 kilometers with a sensitivity value of 35.7% and a specificity value of 96.2% (p < 0.001, AUC = 0.684, 95% CI = 0.613-0.755). Based on these findings, EMS response time and distance from the emergency call receiving location to the collapse location were divided into two groups each.

**Figure 6:**
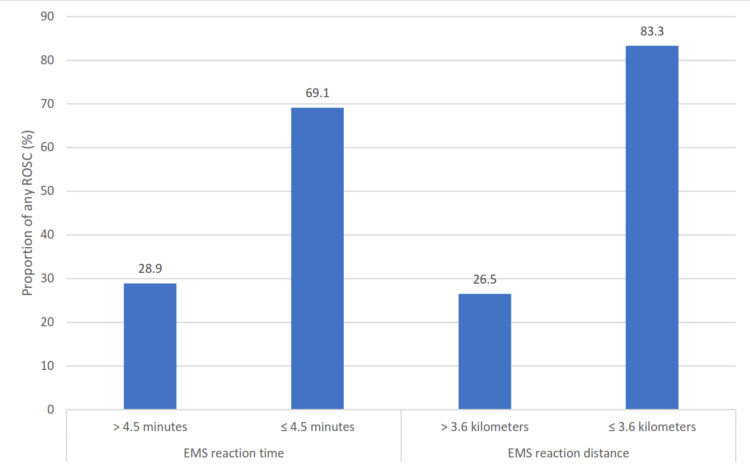
Any ROSC occurrence in different EMS response time and EMS response distance groups. ROSC: return of spontaneous circulation; EMS: emergency medical service.

Comparison of the newly defined groups of the EMS response time and the distance from the emergency call receiving location to the collapse location with the occurrence of any ROSC showed a significant difference (χ^2^(1) = 42.523, p < 0.001 and χ^2^(1) = 43.491, p < 0.001, respectively) (Figure 6).

## Discussion

This study examined various parameters during the pre-hospital management of patients with cardiac cause-OHCA who received bystander CPR before the intervention by the EMS team. It also assessed the impact of these main parameters on positive pre-hospital outcomes, including shockable initial heart rhythm, any ROSC, and survival to hospital admission.

In our sample, cardiac causes were identified in 64.2% of registered OHCA cases. This percentage aligns with the findings from similar studies, suggesting that cardiac causes contribute to approximately 60-70% of OHCA incidents [4,6]. This consistency indicates a similarity in data collection methodologies across various studies. Regional variations in Serbia have been previously noted and were likely influenced by the specific methodologies used to observe and define the factors leading to OHCA [7-10]. Data on the etiology of cardiac arrest gathered from observational EuReCa_Serbia reports have shown cardiac causes to range from 50.0% to 83.8% of cases [3,7-11], which is similar to the findings of the EuReCa_One and EuReCa_Two studies, showing the prevalence of cardiac etiology ranging from 54.8% to 100.0% [1,2]. Likewise, other European studies have reported cardiac etiology rates between 54.8% and 100.0% [1,2,12-14].

Due to the differing initial treatment approaches for cardiac-cause and non-cardiac-cause OHCA, the interpretation of data serving as the foundation for the initial OHCA treatment strategy is complex. Post-mortem experimental studies analyzing OHCA cases with confirmed cardiac etiology have revealed that cardiac causes are present to a significantly lesser extent than presumed. These findings raise questions regarding the accuracy of many epidemiological findings on OHCA causes [15,16].

As an example, a Japanese study conducting detailed examinations of OHCA patients, including perimortem computed tomography, found a cardiac etiology in 37.5% of cases [15]. A similar autopsy study from Australia found a cardiac etiology in only 30.8% of young adults with presumed cardiac-cause OHCA [16]. These findings suggest that in the majority of OHCA cases overall and in a significant percentage of presumed cardiac-cause OHCA cases, there is no pathoanatomical substrate supporting the idea that cardiac disease is the predominant etiological factor for OHCA. Therefore, it can also be presumed that some non-morphological conditions may contribute to the sudden cessation of cardiac function, including various types of electrical phenomena that may occur as a result of different mechanisms, such as metabolic and electrolyte-related disturbances, already proven to be present in a quarter of OHCA patients [17]. Several other publications highlighted the critical role of arrhythmias in causing OHCA and stressed the importance of rapid intervention to improve survival outcomes [18-21]. This challenged the existing paradigm that acute myocardial infarction is the primary cause of cardiac arrest and raised questions about the data structure where the majority of cardiac arrest cases are linked to an underlying cardiac disease, contrasting the findings from autopsy-based studies. This complexity in understanding the real etiology of OHCA remains an enigma and may influence the strategy of OHCA management.

The inclusion of bystanders in applying CPR measures is considered a factor of paramount importance for positive OHCA outcomes. In this current EuReCa_Serbia study, we observed 7.7% of all registered cardiac-cause OHCA cases where bystanders initiated any CPR measures. The majority of other European studies report a similar percentage of inclusion of bystanders in applying CPR measures. The EuReCa_One study reports an overall average of 47.4%, a median of country values of 50.0%, and a range of country values of 6.3-78.0% [1]. The same values reported by the EuReCa_Two study were 58.0%, 57.6%, and 13.0-82.6%, respectively [2]. The European Resuscitation Council Guidelines 2021 on the epidemiology of cardiac arrest in Europe reported that the rate of bystander CPR varies between and within countries, with an average value of 58% and a range of 13-83% [22]. EuReCa_Serbia findings also point to geographical differences within the territory of the Republic of Serbia [23-25].

One of the factors influencing a positive outcome in OHCA is the patient's age. We observed the median patient age in cases where bystander CPR was initiated to be 65 years, similar to other studies that have reported a median/mean patient age of 74.1 years in Australia [26], 66 years in the CARES registry [27], and 61.5-64.7 years in a local US-based study [28].

The residence was the event location in 64.3% of bystander-initiated CPR cases of cardiac-cause OHCA. This factor has also been shown as a very important predictor of shockable initial heart rhythm and survival to admission, which was observed previously in the literature [29].

All of these findings are in accordance with some of the previously published European studies. The EuReCa_Serbia study also noted variations in different time periods and geographical regions, similar to findings from other studies [1-3,11,22,24,25].

In this study, CCO CPR was provided in 38.6%, while full compression and ventilation CPR measures in 35.0%, a finding comparable to the results previously published in a 2023 meta-analysis dealing with this type of bystander CPR-related data [30].

In our study, the shockable first recorded rhythm was observed in 39.8%. Similar results were also observed by the EuReCa_Two study, which reported that the overall average value of detecting shockable initial heart rhythm in CPR-attempted OHCA cases was 20.2%, with the median country value of 19.2%, and the range of 11.4-36.8% of cases [2]. In our analysis, DC shock was delivered in 23.1%, while any ROSC was observed in 34.2%.

Our study identified the patient's age, patient’s residence, and public building as the collapse location as factors significantly associated with the occurrence of a shockable initial heart rhythm, any ROSC, and survival to hospital admission. Factors significantly associated with a shockable initial heart rhythm and any ROSC included whether bystanders applied compression-only CPR or full CPR, and the delivery of DC shock. Additionally, the patient's sex and sports facility as the collapse location were additional factors significantly associated with the occurrence of an initial shockable heart rhythm, while the population size of the sampling region and the street as the location of the collapse were additional factors significantly associated with survival to hospital admission. Most of the previously published studies analyzing cardiac-cause OHCA cases examined survival to hospital discharge as the main outcome [31,32].

Previous studies have confirmed the impact of the time EMS teams require to reach the patient and subsequently conduct the transfer to the nearest hospital, as well as the distance traveled from receiving the emergency call to admission to the ED ward [33-37]. Investigating these time intervals and distances is of crucial importance in identifying remote areas in different urban and rural settings. Remote areas are geographic zones where adequate medical attention for a certain medical condition and the served population is not guaranteed. Given this definition, there is a strong need to determine such zones to ensure optimal reachability to all OHCA patients.

In this study, we observed the association of EMS response time and the interval between the emergency call and arrival at the OHCA location with a shockable initial rhythm (presented with ventricular fibrillation) and any ROSC, but not with survival to hospital admission. This emphasizes the possibility of other factors influencing survival to hospital admission present after achieving any ROSC (e.g., applied pharmacotherapy).

ROC analysis applied in the present study showed an EMS response time cut-off value of 4.5 minutes. Additionally, we observed a 50% cumulative proportion of any ROSC after four minutes and 75% after eight minutes of EMS response time. This time point aligns with the preferred end-time for achieving any ROSC in several European countries. For instance, a study by the German Resuscitation Council noted that reaching the patient at the OHCA location within eight minutes in regions covered by EMS systems with response time reliability higher than 70% occurred in 70.4-95.5% of cases [33].

Studies by other authors have also analyzed these parameters and their influence on outcomes in OHCA patients, showing an association of shorter EMS response time with improved survival rates and better neurological outcomes in OHCA patients. A study published in 2011 found that reducing response time by just one minute can improve the odds of survival by 24% [38]. In the United States, where the EMS system is organized via many different EMS agencies, there is a significant variation in survival rates after OHCA between different agencies. A 2018 study showed that factors such as response time, initial heart rhythm, and whether the arrest was witnessed play crucial roles, and agencies with faster response times generally report higher survival rates [34]. A meta-analysis published in 2015 explained that studies indicate an association between shorter response times with higher rates of ROSC. For example, response times within four minutes were associated with improved ROSC rates, survival to hospital discharge, and one-year survival [39].

Previous studies have shown not only the influence of EMS response time on CPR success rate [40], but also on CPR initiation rate [41], a very important predictor of OHCA survival, which is also influenced by several other factors, including patient age [42], patient sex [43], OHCA etiology [44], OHCA location [45], and time period during the day [46].

In this study, we also examined the distance covered during the EMS response time to pinpoint the threshold where urban remote areas begin. Notably, we found no instances of successful resuscitation, indicated by any ROSC, when this distance exceeded 11.2 km. The significance of distance has been highlighted in prior research [36]. However, there is limited literature exploring distance as a potential factor affecting outcomes in OHCA patients. This scarcity of studies may stem from the diverse geographical landscapes across different regions.

A study published in the Journal of the American Heart Association found that longer distances to EMS were associated with lower survival rates. Specifically, for every kilometer increase in distance, the odds of survival decreased by 8% [37].

Another study published in 2020 analyzing response time and distance indicated that shorter distances to EMS facilities correlate with faster response times, which are crucial for improving survival rates and neurological outcomes. This study highlighted that response times within four minutes significantly increased the chances of ROSC and survival to hospital discharge [35].

A spatial analysis study published in 2023 identified significant geographical disparities in OHCA outcomes. Regions with greater distances to EMS facilities exhibited lower survival rates, underscoring the necessity for tailored interventions in these areas [47].

Also, previous studies have shown that OHCA patients in urban areas generally have better outcomes compared to those in rural areas, primarily due to shorter distances to EMS and quicker response times. This was supported by findings in a systematic review and meta-analysis published in 2022 [20].

In emergency medicine, remote areas are geographic regions where people have insufficient expertise or resources to treat emergency cases. It is often characterized by a combination of low population size and high geographic remoteness [48]. One population includes both urban and rural areas. Similar to many other countries, the Serbian healthcare system does not distribute mobile teams and resources evenly, instead focusing on centralization in urban regions. This organizational structure can impact not only the urban-rural dynamics but also within urban regions themselves. Our study revealed response limitations in terms of time intervals and distances in the cities included in this research, which may be attributed to traffic-related factors.

Additionally, some previous reports show that healthcare professionals have insufficient knowledge about the importance of time on survival improvement in OHCA cases [49], as this study confirmed. This emphasizes the need for education and increasing awareness about time and distance as factors of high importance in OHCA survival. Similarly, regardless of the fact that this analysis was conducted on a group of patients who already received bystander CPR, bystander inclusion in providing CPR measures still remains an irreplaceable factor influencing the survival of OHCA patients.

Although this study has a prospective randomized design, its observational nature limits its ability to report specific causalities. Another limitation is the limited spectrum of information collected, including population density, urban/rural differences, and pharmacotherapy given prior to hospital admission, which could not be bypassed due to the fact that the study is based on an already established and registered trial protocol. A difference in data collection consistency before and during hospitalization of OHCA patients is an additional shortcoming, limiting this study’s potential to directly assess the predictive value of pre-hospital data on survival to hospital discharge with the strength of results similar to those focusing on pre-hospital outcomes.

## Conclusions

This study showed that in cases of cardiac-cause OHCA in adults where bystanders initiate CPR measures, location, patient sex, and bystander CPR type play an important role in predicting a shockable initial heart rhythm, while sex loses its potential for predicting any ROSC. The main predictors of survival to hospital admission are population size and OHCA location. Both shorter EMS response time and a shorter distance are significantly associated with a higher rate of a shockable initial rhythm and any ROSC, while not having an influence on admission survival.

This study’s observations can contribute to a better organizational structure of healthcare systems as well as a more precise definition of treatment procedures served by remote EMS teams. The study also highlights the need for EMS teams to be dispersed across underserved territories.
